# A Wnt10a-Notch signaling axis controls Hertwig’s epithelial root sheath cell behaviors during root furcation patterning

**DOI:** 10.1038/s41368-024-00288-x

**Published:** 2024-03-13

**Authors:** Kai Sun, Miao Yu, Jiayu Wang, Hu Zhao, Haochen Liu, Hailan Feng, Yang Liu, Dong Han

**Affiliations:** 1 Department of Prosthodontics, Peking University School and Hospital of Stomatology & National Center for Stomatology & National Clinical Research Center for Oral Diseases & National Engineering Research Center of Oral Biomaterials and Digital Medical Devices, Beijing, China; 2 Chinese Institute for Brain Research, Beijing, China

**Keywords:** Cell proliferation, Differentiation

## Abstract

Human with bi-allelic *WNT10A* mutations and epithelial *Wnt10a* knockout mice present enlarged pulp chamber and apical displacement of the root furcation of multi-rooted teeth, known as taurodontism; thus, indicating the critical role of *Wnt10a* in tooth root morphogenesis. However, the endogenous mechanism by which epithelial *Wnt10a* regulates Hertwig’s epithelial root sheath (HERS) cellular behaviors and contributes to root furcation patterning remains unclear. In this study, we found that HERS in the presumptive root furcating region failed to elongate at an appropriate horizontal level in *K14-Cre;Wnt10a*^*fl/fl*^ mice from post-natal day 0.5 (PN0.5) to PN4.5. EdU assays and immunofluorescent staining of cyclin D1 revealed significantly decreased proliferation activity of inner enamel epithelial (IEE) cells of HERS in *K14-Cre;Wnt10a*^*fl/fl*^ mice at PN2.5 and PN3.5. Immunofluorescent staining of E-Cadherin and acetyl-α-Tubulin demonstrated that the IEE cells of HERS tended to divide perpendicularly to the horizontal plane, which impaired the horizontal extension of HERS in the presumptive root furcating region of *K14-Cre;Wnt10a*^*fl/fl*^ mice. RNA-seq and immunofluorescence showed that the expressions of Jag1 and Notch2 were downregulated in IEE cells of HERS in *K14-Cre;Wnt10a*^*fl/fl*^ mice. Furthermore, after activation of Notch signaling in *K14-Cre;Wnt10a*^*fl/fl*^ molars by Notch2 adenovirus and kidney capsule grafts, the root furcation defect was partially rescued. Taken together, our study demonstrates that an epithelial Wnt10a-Notch signaling axis is crucial for modulating HERS cell proper proliferation and horizontal-oriented division during tooth root furcation morphogenesis.

## Introduction

The tooth root is the part of the tooth that is embedded in the jawbone and provides structural support and stability.^1^ Multirooted teeth provide stronger anchorage and better resistance of chewing and biting forces than single-rooted teeth.^2^ Tooth root patterning, which commences after the completion of crown formation, depends on crucial interactions between dental epithelial and mesenchymal cells.^3^ So far, a series of signals from dental mesenchyme cells, including GLI family zinc finger 1 (GLI1), nuclear factor I C (NFIC), fibroblast growth factors (FGFs), transforming growth factor-β (TGF-β), and bone morphogenetic proteins (BMPs) have been identified as key regulatory factors in tooth root formation.^3–7^ By contrast, although some epithelial signals such as sonic hedgehog (SHH), ectodysplasin A (EDA) and interferon regulatory factor 6 (IRF6), have been proved to regulate tooth root development, the specific roles of molecules expressed in the dental epithelium and their related signaling networks involved in root patterning remain largely unknown.^3,8,9^

The Hertwig’s epithelial root sheath (HERS) is a critical structure that guides tooth root patterning.^10,11^ HERS is derived from the apical extension of the cervical loop in the enamel organ at the beginning of root development and consists of two cell layers; the inner and outer enamel epithelium (IEE and OEE, respectively).^12^ The boundaries between the HERS and the crown epithelium can be demarcated based on two principles: the dental epithelium cells in HERS are cuboidal in shape, and the dental papilla mesenchyme adjacent to the newly formed HERS has not yet differentiate into odontoblasts.^3,13^ Directional horizontal elongation of the HERS determines the shape and position of the furcation in multirooted teeth.^14^ Failure of HERS elongation at the proper horizontal level can lead to taurodontism, which is characterized by the absence of or apically located pulp floor and pulp chamber enlargement.^15^ The defective phenotypes of root furcation have been investigated in mutant animal models; however, studies have been limited to morphological investigations.^8,9,16,17^ The precise cellular and molecular mechanisms of root furcation defects have not been systematically elucidated.

*Wnt10a* (wingless-type MMTV integration site family, member 10 A), a ligand in canonical Wnt signaling, plays a critical role in regulating tooth development.^18^ In the early stage of tooth development, *Wnt10a* is first expressed in dental placode and later in primary enamel knot, and is speculated to function in tooth development initiation and crown patterning.^19,20^ Then, the expression of *Wnt10a* starts to shift from secondary enamel knot to the underlying mesenchymal cells at early bell stage.^20^ During post-natal stage, *Wnt10a* expresses in odontoblast cell layer and functions as a key molecule for dentinogenesis during tooth root development.^20^ Noteworthily, although the epithelial *Wnt10a* signal is comparatively weaker than that in adjacent odontoblasts, we previously suggested that epithelial knockout of *Wnt10a* caused taurodontism in mice, and that epithelial *Wnt10a* might regulate the well-organized proliferation of dental mesenchymal cells in the presumptive root furcating region and be a key factor in root furcation patterning.^18^ These data indicate the critical role of epithelial *Wnt10a* in tooth root furcation patterning.

The Notch signaling pathway is evolutionarily conserved and is involved in the development of multiple organs.^21–24^ In addition, it is involved in the regulation of tooth shape patterning.^23^ Notch signaling depression in mice results in differential Bmp4 expression and apoptosis in the enamel knot, which leads to crown shape malformation, typically by additional cusp formation.^23^ Membrane receptors of Notch signaling, Notch1 and Notch2, are expressed in the mouse embryonic cervical loop on day 19 (E19), shortly before root development begins.^25^ In addition, Notch signaling ligands, Jagged1 (Jag1) and Jagged2 (Jag2), are expressed in IEE cells during the root development stage of postnatal molars.^25^ However, the molecular mechanism by which Notch signaling coordinate with epithelial *Wnt10a* in regulating HERS cellular behaviors and root furcation patterning remains unclear.

Therefore, In the present study, we systematically analyzed the histological defects of HERS as well as alterations in the behaviors of IEE and OEE cells in the presumptive root furcating region of *K14-Cre;Wnt10a*^*fl/fl*^ mice during the early stage of postnatal tooth furcation development, in order to elucidate the functional significance of epithelial *Wnt10a* in modulating HERS cell behaviors during root furcation morphogenesis.

## Results

### Ablation of epithelial *Wnt10a* impaired the horizontal elongation of HERS cells

**Fig. 1:**
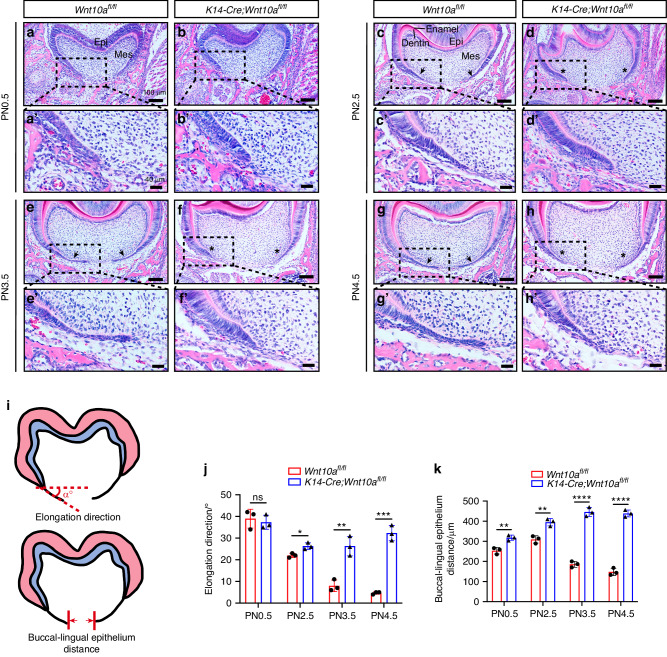
Comparison of tooth furcation histology during early stage postnatal root development of the mandibular first molar. **a**–**h** Coronal images of H&E-stained mandibular first molars (M1) from *Wnt10a*^*fl/fl*^ and *K14-Cre;Wnt10a*^*fl/fl*^ mice at PN0.5, PN2.5, PN3.5, and PN4.5. Black arrows indicate HERS formation in *Wnt10a*^*fl/fl*^ mice, and black asterisks indicate developmental failure of the furcation region in *K14-Cre;Wnt10a*^*fl/fl*^ mice. Epi, epithelium; Mes, mesenchyme. Scale bars: 100 μm. **a’**–**h’** Higher magnifications of the lingual furcation region. Scale bars: 40 μm. **i** Schematic diagrams of measuring methods of the elongation direction and the buccal-lingual epithelium distance. **j**, **k** Bar graphs depicting HERS elongation direction (**j**), and buccal-lingual distance (**k**) in *Wnt10a*^*fl/fl*^ and *K14-Cre;Wnt10a*^*fl/fl*^ mice. *n* = 3 per group. Data are presented as mean ± SD. ns not significant, *P < 0.05, **P < 0.01, ***P < 0.001, ****P < 0.000 1

To observe the initial time point of root furcation development, we performed H&E staining on coronal sections of the first mandibular molars from PN0.5 to PN4.5 *Wnt10a*^*fl/fl*^ and *K14-Cre;Wnt10a*^*fl/fl*^ mice. Specifically, we analyzed the horizontal distance between the buccal and lingual sides of the cervical loop (or HERS) as well as the elongation direction of lingual cervical loop (or HERS) within the presumptive root furcating region. At PN0.5, no significant difference was observed regarding to the elongation direction of cervical loop between the two groups, but the buccal-lingual distance was mildly larger in *K14-Cre;Wnt10a*^*fl/fl*^ mice (Fig. 1a-b, a’-b’, j, k). Noteworthily, at PN2.5, the HERS with bilayered structure started to be observed at the anterior end of cervical loop in *Wnt10a*^*fl/fl*^ mice (Fig. 1c, c’). These results indicate that PN2.5 is the initial time point of HERS formation during root furcation development. Meanwhile, PN2.5 *K14-Cre;Wnt10a*^*fl/fl*^ mice showed significant increased angle between HERS elongation and the horizontal plane, as well as larger buccal-lingual HERS distance (Fig. 1d, d’, j, k). At PN3.5 and PN4.5, the buccal-lingual HERS distance was significantly longer in *K14-Cre;Wnt10a*^*fl/fl*^ mice than which of *Wnt10a*^*fl/fl*^ mice (Fig. 1e–h, e’–h’, k). Moreover, the angle between HERS elongation direction and the horizontal plane was <10° in *Wnt10a*^*fl/fl*^ mice (Fig. 1e’, g’, j). In contrast, in *K14-Cre;Wnt10a*^*fl/fl*^ mice, the deficiency of epithelial *Wnt10a* significantly increased the angle between the HERS elongation direction and the horizontal plane to >20°(Fig. 1f’, h’, j), and resulted in HERS vertical elongation. These results demonstrate that ablation of epithelial *Wnt10a* impaired the horizontal elongation of HERS cells.

### Ablation of epithelial *Wnt10a* inhibited the proliferation activity of IEE cells in HERS

**Fig. 2:**
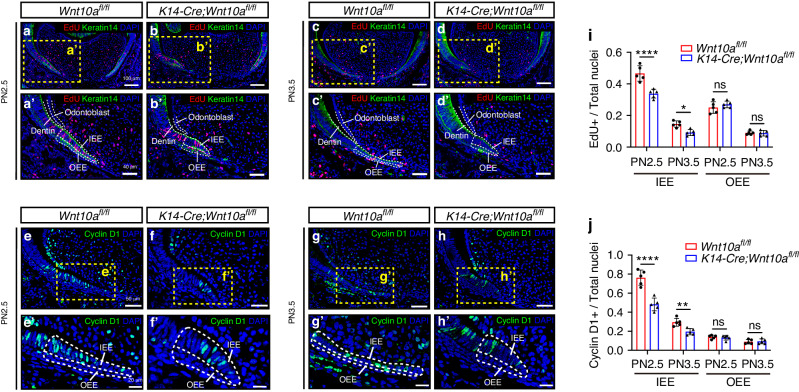
Decreased proliferation activity and cyclin D1 expression in dental epithelial cells of *K14-Cre;Wnt10a*^*fl/fl*^ mice. **a–d** Co-localization of anti-Keratin14 (green), EdU (red), and DAPI (blue) shows the proliferation activity of dental epithelial cells in the lingual root furcation region of M1 from *Wnt10a*^*fl/fl*^ and *K14-Cre;Wnt10a*^*fl/fl*^ mice at PN2.5 and PN3.5. Scale bars: 100 μm. **a’–d’** Higher magnification of the yellow dashed boxes in (**a**–**d**). White dashed lines outline IEE and OEE cells in the root furcation region used for quantitative analysis. Scale bars: 40 μm. **e**–**h** Co-localization of anti-cyclin D1 (green) and DAPI (blue) shows cyclin D1 expression in IEE and OEE cells within the root furcation region of M1 from *Wnt10a*^*fl/fl*^ and *K14-Cre;Wnt10a*^*fl/fl*^ mice at PN2.5 and PN3.5. Scale bar: 50 μm. **e’**–**h’** Higher magnifications of the yellow dashed boxes in (**e**–**h**). White dashed lines outline IEE and OEE cells in the root furcation region used for quantitative analysis. Scale bars: 20 μm. **i** Ratios of EdU^+^/ DAPI^+^ cells representing IEE or OEE cell proliferation rates. *n* = 5 per group. **j** Ratios of cyclin D1^+^/ DAPI^+^ cells representing cyclin D1 expression in IEE or OEE cells. *n* = 5 per group. Data are presented as mean ± SD. ns not significant, *P < 0.05, **P < 0.01, ****P < 0.000 1

To investigate the cellular mechanism responsible for the root furcation defect in *K14-Cre;Wnt10a*^*fl/fl*^ mice, we used the 5-ethynyl-2′-deoxyuridine (EdU) proliferation assay, immunofluorescence of cyclin D1, and terminal deoxynucleotidyl transferase dUTP nick end labeling (TUNEL) assay to detect the proliferation and apoptosis of HERS cells in the presumptive root furcating region (Fig. 2a–d, a’–d’, i; Supplementary Fig. 2). Quantitative analysis using EdU- and DAPI-stained nuclei revealed that the proliferation of IEE cells in HERS was significantly decreased in *K14-Cre;Wnt10a*^*fl/fl*^ mice at PN2.5 and PN3.5 (Fig. 2i); however, no significant difference of the proliferation of OEE cells in HERS was observed at these stages (Fig. 2i). Consistent with the EdU proliferation assay, the expression of cyclin D1 was also significantly reduced in IEE cells of HERS in *K14-Cre;Wnt10a*^*fl/fl*^ mice at PN2.5 and PN3.5 (Fig. 2e–h, e’–h’, j); but no significant difference of the expression of cyclin D1 was observed in OEE cells at these stages (Fig. 2j). In addition, no apoptotic signals were detected in HERS cells of the presumptive root furcating region in each group (Supplementary Fig. 2). These results demonstrated that epithelial *Wnt10a* knockout impairs the proliferative activity of IEE cells in HERS during root furcation development.

### Ablation of epithelial *Wnt10a* resulted in vertically oriented division of IEE cells in HERS

**Fig. 3:**
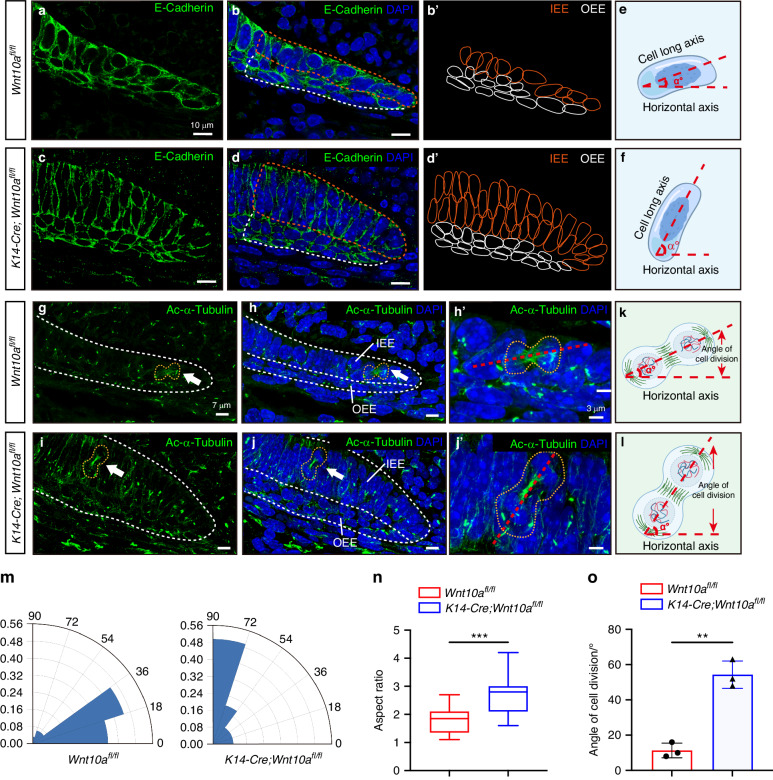
Altered cell long axis and misoriented cell division of IEE cells in *K14-Cre;Wnt10a*^*fl/fl*^ mice. **a**–**d** Co-localization of anti-E-cadherin (green) and DAPI (blue) shows the morphology of HERS cells in the lingual side of the root furcation region of M1 from *Wnt10a*^*fl/fl*^ and *K14-Cre;Wnt10a*^*fl/fl*^ mice at PN2.5. Orange and white dashed lines outline the IEE and OEE cells, respectively. Scale bars: 10 μm. **b’**, **d’** Diagrams of IEE and OEE cell shapes in M1 from *Wnt10a*^*fl/fl*^ (**b’**) and *K14-Cre;Wnt10a*^*fl/fl*^ mice (**d’**), based on the staining results in (**a**–**d**). **e**, **f** Schematic diagrams of α angle measurement between cell long and horizontal axes. **g**–**j** Co-localization of anti-Ac-α-Tubulin (green) and DAPI (blue) shows IEE cell division direction in the root furcation region of M1 from *Wnt10a*^*fl/fl*^ and *K14-Cre;Wnt10a*^*fl/fl*^ mice at PN2.5. White dashed lines outline the IEE and OEE cells. The orange dashed lines outline the dividing IEE cells. Scale bars: 7 μm. **h’**, **j’** Higher magnifications of the cells marked by orange dashed lines in (**h**) and (**j**). Red dashed lines indicate the division direction. Scale bars: 3 μm. **k**, **l** Schematic diagrams of the α angle between the direction of cell division and the measured horizontal axis. **m** Radial histogram quantification of the α angle between the cell long and horizontal axes based on (**b’**, **d’**). **n** Quantification of the aspect ratio of IEE cells in (**b’**, **d’**). **o** Quantification analysis of the α angle based on (**h’**, **j’**). *n* = 3 per group. Data are presented as mean ± SD. ns not significant, **P < 0.01, ***P < 0.001

The orientation of cell division is an important cellular behavior that determines the direction of cell growth and tissue morphogenesis.^26^ The long-axis of a cell can predict the orientation of cell division based on the long-axis rule introduced by Oscar Hertwig in 1884.^27^ Therefore, we used super-resolution microscopy to observe the direction of cell division in lingual HERS cells in presumptive root furcating region. E-cadherin staining (Fig. 3a–d) and outlines of IEE and OEE cells (Fig. 3b’, d’) showed the profile of IEE and OEE cells more clearly. Analysis of the cell long-axis showed that ~80% of IEE cells in presumptive root furcating region had a minor angle (<36°) between the cell long-axis and the horizontal plane at PN2.5 *Wnt10a*^*fl/fl*^ mice (Fig. 3e, f, m). In contrast, at PN2.5 *K14-Cre;Wnt10a*^*fl/fl*^ mice, over 70% of the IEE cells in presumptive root furcating region had an angle >54° (Fig. 3e, f, m), implying a vertical tendency of IEE cell division. Analysis of cell shape showed that in the furcation region of *K14-Cre;Wnt10a*^*fl/fl*^ mice, the aspect ratio (major axis length versus minor axis length) of IEE cells in the HERS was larger, indicating the IEE cells were columnar-shaped rather than oval-shaped in *Wnt10a*^*fl/fl*^ mice (Fig. 3n). Furthermore, through immunofluorescence staining of acetyl-α-Tubulin, a known mitotic spindle marker used to observe cell mitosis, we determined the cell division direction of IEE cells (Fig. 3g–j, h’, j’). Analysis of the mitotic spindle orientation showed that the angle between the direction of cell division of IEE cells and the horizontal plane in *Wnt10a*^*fl/fl*^ mice was significantly smaller than that in *K14-Cre;Wnt10a*^*fl/fl*^ mice in presumptive root furcating region at PN2.5 (Fig. 3k, l, o). These results confirmed that the ablation of epithelial *Wnt10a* resulted in a vertically oriented tendency of IEE cell mitosis at the initial time point of HERS formation during root furcation development, which impaired the horizontal elongation of HERS.

### Ablation of epithelial *Wnt10a* suppressed Notch signaling in IEE cells

**Fig. 4:**
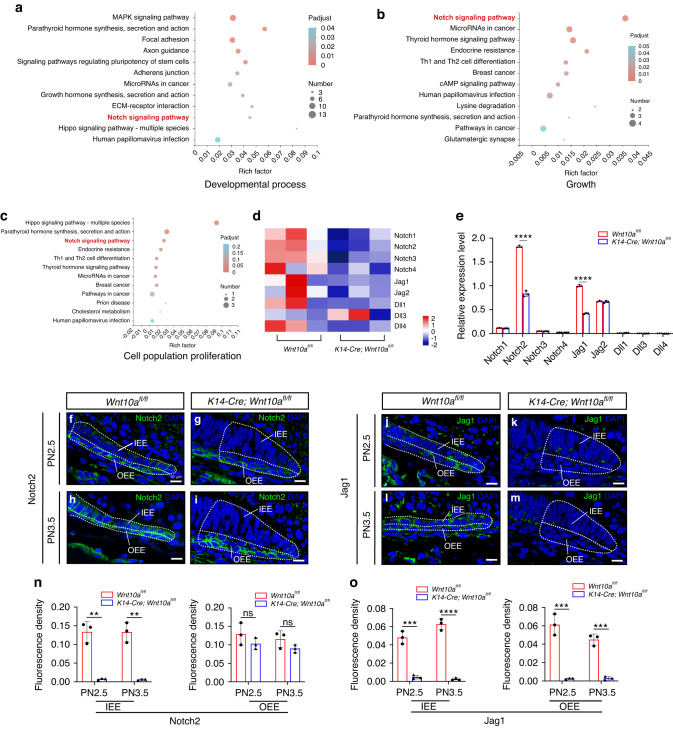
Loss of epithelial *Wnt10a* compromises Notch signaling in IEE cells. **a**–**c** Bubble maps of the KEGG pathway enrichment analysis for differentially expressed gene sets, including developmental process, growth, and cell population proliferation. The size of the bubbles represents the number of differentially expressed genes, and the color shade depends on the adjusted *P*-value of each term. Bold red font indicates the Notch signaling pathway. **d** Heatmap showing the relative expression levels of critical ligands and receptors in the Notch signaling pathway of the M1 dental epithelium from *Wnt10a*^*fl/fl*^ and *K14-Cre;Wnt10a*^*fl/fl*^ mice at PN2.5. **e** RT-qPCR results for critical ligands and receptors in the Notch signaling pathway in the M1 dental epithelium of *Wnt10a*^*fl/fl*^ and *K14-Cre;Wnt10a*^*fl/fl*^ mice at PN2.5. Jag1 expression in *Wnt10a*^*fl/fl*^ samples was set to 1. *n* = 3 per group. **f–i** Super-resolution imaging analysis of Notch2 expression patterns in dental epithelium within the lingual furcation region at PN2.5 and PN3.5. White dashed lines outline IEE and OEE cells in the root furcation region. Scale bars: 10μm. **j**–**m** Super-resolution imaging analysis of Jag1 expression patterns in dental epithelium within the lingual furcation region at PN2.5 and PN3.5. White dashed lines outline IEE and OEE cells in the root furcation region. Scale bars: 10 μm. **n**, **o** Fluorescence density analysis of Notch2 (**n**) and Jag1 (**o**) in IEE or OEE cells in (**f**–**m**). *n* = 3 per group. Data are presented as mean ± SD. ns not significant, **P < 0.01, ***P < 0.001, ****P < 0.000 1

As PN2.5 is an essential stage for initiating HERS formation, critical downstream molecules that control furcation patterning may begin to be expressed at this point. Therefore, we performed RNA-seq analysis of epithelial tissue from the mandibular first molars of *Wnt10a*^*fl/fl*^ and *K14-Cre;Wnt10a*^*fl/fl*^ mice at PN2.5. First, we identified 284 differentially expressed genes and chose three gene sets, including those involved in the developmental process, growth, and cell population proliferation (Fig. 4a–c). Using KEGG analysis, we found that the Notch signaling pathway, which has been reported to be involved in tooth morphological development,^25^ was highly enriched in all chosen gene sets (Fig. 4a–c). Notch2 is one of the membrane receptors of Notch signaling and can release its intracellular domain (N2ICD) to activate Notch signaling when bound with Jag1.^28^ The heatmap and qRT-PCR of core molecules in Notch signaling demonstrated that Notch2 and Jag1 were both significantly downregulated in molar epithelium of PN2.5 *K14-Cre;Wnt10a*^*fl/fl*^ mice (Fig. 4d, e).

Subsequently, we employed immunofluorescence staining to detect the expression patterns of Notch2 and Jag1 in vivo. Notch2 was expressed in both OEE and IEE cells of *Wnt10a*^*fl/fl*^ mice in HERS within presumptive root furcating region at PN2.5 and PN3.5 (Fig. 4f, h), whereas it was only observed in OEE cells when epithelial *Wnt10a* was ablated (Fig. 4g, i). Fluorescence density analysis showed that Notch2 expression significantly decreased in IEE cells of *K14-Cre;Wnt10a*^*fl/fl*^ mice, whereas there was no statistically significant difference in OEE cells between the two groups (Fig. 4n). Moreover, Jag1 was expressed in IEE and OEE cells of *Wnt10a*^*fl/fl*^ mice in HERS within presumptive root furcating region at PN2.5 and PN3.5 (Fig. 4j, l), but it was not detected in HERS cells of *K14-Cre;Wnt10a*^*fl/fl*^ mice (Fig. 4k, m). Fluorescence density analysis showed that Jag1 expression significantly decreased in both IEE and OEE cells of *K14-Cre;Wnt10a*^*fl/fl*^ mice (Fig. 4o). These results indicate that epithelial deletion of *Wnt10a* results in decreased Notch2 and Jag1 expression in HERS IEE cells, which might suppress the Notch signaling transduction.

### Overexpression of Notch2 partially rescued root furcation defects of *K14-cre;Wnt10a*^*fl/fl*^ mice

To further test whether epithelial Notch signaling acts as a downstream target of *Wnt10a* in root furcation patterning, in vivo rescue experiments were performed using adenovirus-mediated Notch2 overexpression in PN0.5 mandibular first molars. After 24 h of adenovirus infection, fluorescence microscopy detected strong EGFP signals in dental epithelium within the cervical part of PN0.5 mandibular first molars (Supplementary Fig. 3a–d, f). In addition, western blot analysis showed that, when compared with the Ad-Control group, N2ICD expression was upregulated fivefold in Ad-N2ICD-Over group (Supplementary Fig. 3e), indicating that adenovirus infection and Notch2 overexpression were successful in PN0.5 mandibular first molars.

**Fig. 5:**
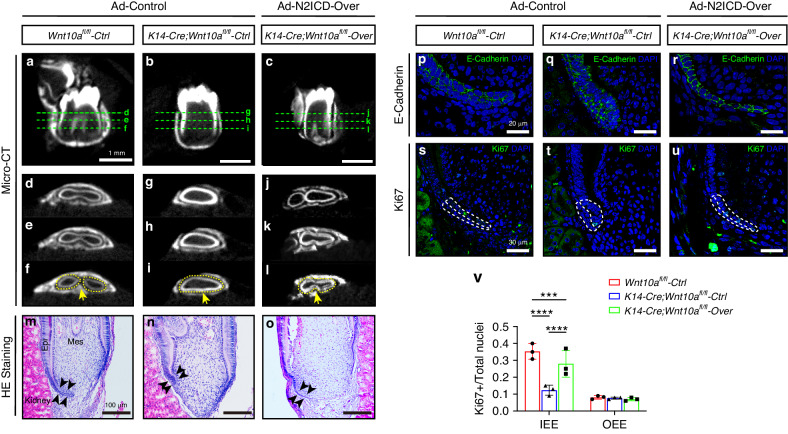
Overexpression of Notch2 partially rescues root furcation developmental defects of *K14-Cre;Wnt10a*^*fl/fl*^ mice. **a**–**l** Micro-CT images of *Wnt10a*^*fl/fl*^ molars infected with Ad-control (**a**, **d**–**f**), *K14-Cre;Wnt10a*^*fl/fl*^ molars infected with Ad-control (**b**, **g**–**i**), or *K14-Cre;Wnt10a*^*fl/fl*^ molars infected with Ad-N2ICD (**c**, **j**–**l**). **d**–**l** Axial views of the upper (**d**, **g**, **j**), middle (**e**, **h**, **k**), and lower (**f**, **i**, **l**) one-third of the molar roots from the three groups. Arrows indicate the root furcation region, and yellow dashed lines indicate the contour of each root. Scale bars: 1 mm. **m**–**o** HE staining of tooth furcation 3 days after kidney capsule grafts. Black arrowheads indicate the HERS within furcation region. Epi, epithelium; Mes, mesenchyme. Scale bars: 100 μm. **p–r** E-cadherin immunofluorescence staining of tooth furcation 3 days after kidney capsule grafts. Scale bars: 20 μm. **s–u** Ki67 immunofluorescence staining of tooth furcation shows the proliferation activity of HERS cells in the lingual root furcation region 3 days after kidney capsule grafts. White dashed lines outline IEE and OEE cells in the root furcation region. Scale bars: 30 μm. **v** Ratios of Ki67^+^/DAPI^+^ cells representing IEE or OEE cell proliferation rates. *n* = 3 per group. ***P < 0.001, ****P < 0.000 1

After 3 weeks of growth under the kidney capsule, micro-computed tomography (micro-CT) showed that the Ad-Control-infected and Notch2 overexpressed *Wnt10a*^*fl/fl*^ mandibular first molars were all well developed with a normal root furcation shape (Fig. 5a, d–f; Supplementary Fig. 4). In contrast, the Ad-Control-infected *K14-Cre;Wnt10a*^*fl/fl*^ mandibular first molars showed a taurodontic phenotype, with the absence of root furcation (Fig. 5b, g–i). Comparatively, for Notch2 overexpressed *K14-Cre;Wnt10a*^*fl/fl*^ mandibular first molars, an obvious horizontal invagination was observed in the middle region of the molar root in the axial plane (Fig. 5c, j–l). Our results indicated that upregulation of Notch signaling using Notch2 overexpression adenovirus partially corrected the taurodontism phenotype in *K14-Cre;Wnt10a*^*fl/fl*^ mice.

To further confirm the restoration of HERS morphology and proliferation activity regulated by the upregulation of Notch signaling, we conducted morphological analysis. HE staining and E-cadherin immunofluorescence staining showed that, 3 days after kidney capsule grafts, the HERS of furcation region in the Ad-Control-infected *K14-Cre;Wnt10a*^*fl/fl*^ group elongated towards the root apex, with IEE showing a phenomenon of cell stratification and pile-up (Fig. 5n, q). In the Notch2 overexpressed *K14-Cre;Wnt10a*^*fl/fl*^ group, the HERS of furcation region tended to elongate horizontally, and IEE cells formed single layer arrangement (Fig. 5o, r), resembling the IEE cells in Ad-Control-infected *Wnt10a*^*fl/fl*^ group (Fig. 5m, p). Ki67 immunofluorescence staining revealed that the impaired proliferative activities of IEE cells in the HERS of furcation region in the Notch2 overexpressed *K14-Cre;Wnt10a*^*fl/fl*^ group were partially improved (Fig. 5s-v). Our results indicated that upregulation of Notch signaling using Notch2 overexpression adenovirus corrected the elongation direction of HERS in the furcation region from perpendicular to horizontal, and partially improved the proliferation of IEE cells in *K14-Cre;Wnt10a*^*fl/fl*^ mice.

### *Wnt10a* regulates Notch signaling via active-β-catenin in dental epithelial cells

**Fig. 6:**
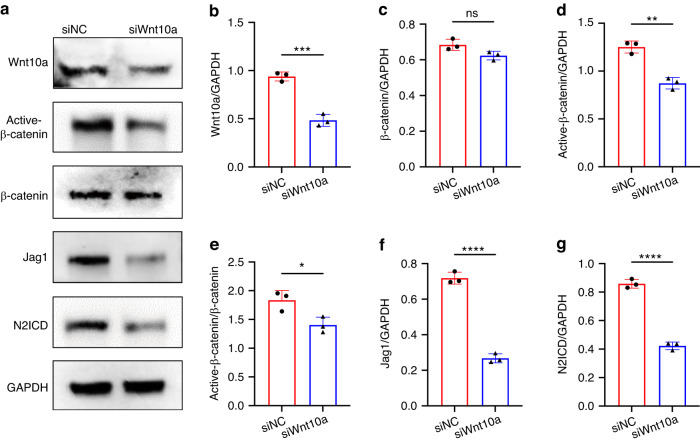
Epithelial *Wnt10a* regulates Notch signaling via active-β-catenin in dental epithelial cells. **a** Western blot analysis of Wnt10a, active-β-catenin, β-catenin, Jag1, N2ICD in *Wnt10a* silenced LS8 cells. **b**–**g** Quantitative analysis of western blot results in (**a**). *n* = 3 per group. ns not significant, *P < 0.05, **P < 0.01, ***P < 0.001, ****P < 0.000 1

To further discuss how epithelial *Wnt10a* regulated the Notch signaling, we utilized siRNA to knockdown the expression of *Wnt10a* in LS8 cell line, a classical model of dental epithelial cell. Consistent with the immunofluorescence staining results, after knockdown of Wnt10a (Fig. 6a, b), the expression levels of N2ICD and Jagged1 in LS8 cells were both decreased (Fig. 6a, f, g). Furthermore, the active level of β-catenin was significantly impaired in siWnt10a group (Fig. 6a, e). These results demonstrated that epithelial *Wnt10a* might regulates upstream Notch signaling via active-β-catenin, which is consistent with previous studies in epithelial cells.^29,30^

## Discussion

**Fig. 7:**
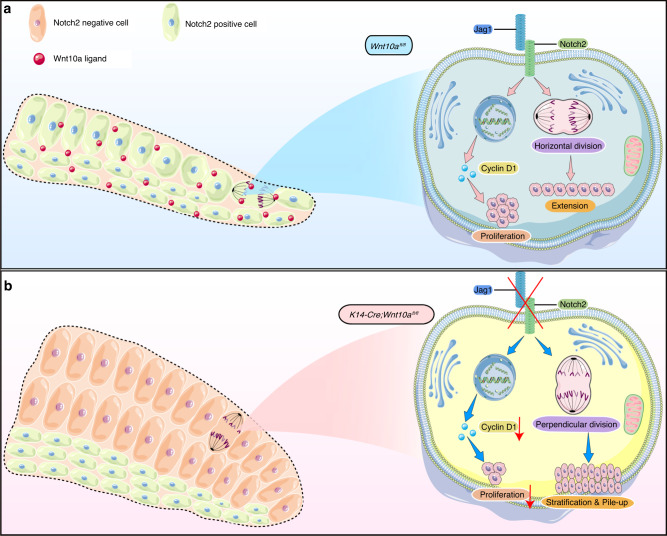
Schematic drawing of the role of epithelial Wnt10a-Notch signaling in regulating root furcation patterning. **a** Epithelial Wnt10a-Notch signaling plays an important role in regulating the horizontal elongation of HERS. Physiologically, Wnt10a-Jag1/Notch2 regulates IEE cell proliferation by tuning the cell cycle to provide extension dynamics, and directly controls the horizontal direction of IEE cell division to determine the direction of HERS extension. **b** Absence of epithelial Wnt10a results in the down-regulation of Jag1/Notch2, which not only disturbs the IEE cell cycle and leads to extension dynamics impairment, but also changes the cell division orientation perpendicularly and results in regional stratification and cell pile-up of IEE cells. Subsequently, the HERS in the root furcation region fails to extend horizontally

A deeper understanding of the mechanisms regulating tooth root patterning can elucidate the common principles that modulate organ morphogenesis. In the present study, we focused on the epithelial endogenous regulatory mechanisms underlying HERS misoriented extension that impaired root furcation formation in multirooted teeth, using an epithelial *Wnt10a* knockout mouse model. We found that absence of epithelial *Wnt10a*, resulted in the down-regulation of Jag1/Notch2, which not only disturbed the IEE cell cycle and led to extension dynamics impairment, but also changed the cell division orientation perpendicularly and resulted in regional stratification and pile-up of IEE cells. Subsequently, the HERS in the root furcation region failed to elongate horizontally (Fig. 7). Our research has, for the first time, confirmed the cascading regulatory effect of the Wnt10a-Notch signaling axis on the oriented cell division and proliferation activity of HERS cells during furcation patterning.

The canonical Wnt signaling plays an important role in tooth root development.^31,32^ *Wnt10a*, a ligand of the canonical Wnt pathway, presents intensive expression in odontoblast cells adjacent to dental epithelium.^20^ A recent study proved that *Wnt10a* induced pupal mesenchyme progenitors residing in furcation region to differentiate into odontoblasts and form a horizontal dentin bridge, whereas inhibition of *Wnt10a* expression disrupted dentin bridge formation and resulted in taurodontism.^33^ In previous study, we found that *Wnt10a* knockout in dental epithelium also caused taurodontism, and proved that epithelial *Wnt10a* regulated the expression of pupal mesenchymal *Wnt4* adjacent to dental epithelium to control root furcation morphogenesis.^18^ In the present study, we focused on the epithelial endogenous mechanisms underlying furcation patterning and found that the ablation of epithelial *Wnt10a* impaired the horizontal elongation of HERS. Furthermore, decreased proliferation activity and vertically oriented division of IEE cells were observed in *K14-Cre;Wnt10a*^*fl/fl*^ mice. Additionally, we also observed that when HERS initiated to guide the furcation formation, columnar-shaped IEE cells transformed to oval-shaped. In contrast, in epithelial-*Wnt10a* deleted mice, the IEE cells sustained their columnar shape. These data suggest that epithelial *Wnt10a* acts as a pivotal signal that orchestrates the cell behaviors of IEE to guide furcation patterning.

In this study, we observed that the cellular behaviors of IEE cells were significantly affected following the knockout of epithelial *Wnt10a* at the initial stage of furcation formation, while the changes in OEE cells were not evident. The role of IEE and OEE cells in the process of tooth crown formation are relatively well-understood,^34,35^ but the functions and cellular behaviors of these two groups of cells are currently unclear in furcation formation. Through transcript detection of *Wnt10a*, we uncovered that *Wnt10a* was highly expressed in the IEE cells of the HERS in furcation region, while it expressed at a lower level in the IEE cells of the HERS in non-furcation region (Supplementary Fig. 5). We speculate that it is the high expression level of *Wnt10a* in IEE that drives the proliferation of IEE cells as well as horizontal-oriented cell division, and determines the patterning of furcation region. In contrast, although *Wnt10a* was physiologically expressed in OEE cells of HERS in furcation region (Supplementary Fig. 5), the cellular behaviors of OEE cells remain unchanged in epithelial *Wnt10a* deleted mice, indicating that *Wnt10a* is redundant in regulation of OEE cell behaviors.

The Notch signaling has been extensively studied to control epithelial organ morphology through regulating cell proliferation.^36,37^ During the development of ciliary body in murine eye, the Notch signaling modulates the differential proliferation activity between epithelium layers, which provides critical dynamics for ciliary body morphogenesis.^37^ In our study, cyclin D1 signals of IEE cells were significantly reduced within the furcation region in *K14-Cre;Wnt10a*^*fl/fl*^ mice. Considering that Notch signaling directly initiates the transcription of cyclin D1 to regulate cell cycle,^38^ we therefore concluded that epithelial *Wnt10a* may regulate IEE cell proliferation through Notch signaling via cyclin D1 to provide the extension dynamics of HERS. On the other hand, the Notch signaling also mediates cell division orientation to guide tissue extension and shape organs correctly.^37^ In Notch-deficient mice kidneys, the division angle between the tubular epithelial cells and the basement membrane altered from parallel to perpendicular, resulting in densely packed regional cells and a stratified epithelium.^36^ In *K14-Cre;Wnt10a*^*fl/fl*^ mice, IEE cells in the furcation region also showed locally packed and stratified phenomena, as well as altered long-axis orientation and mitotic spindle orientation. This supports the notion that Notch signaling may act downstream of epithelial *Wnt10a* in regulating the orientation of epithelial cell division during furcation morphogenesis.

Wnt signaling has been found to orchestrate epithelial cell behavior by mediating Notch signaling in organ morphogenesis.^39^ During *Drosophila* wing primordium patterning, Wnt and Notch signaling functions synergistically to endow epithelial cells with the ability of orientated growth.^40^ In addition, the Wnt-Notch signaling navigates chicken bud extensions to determine feather direction.^39^ In our study, Jag1 and Notch2 were found to be downregulated in HERS cells of *K14-Cre;Wnt10a*^*fl/fl*^ mice by RNA-seq and qRT-PCR analyses. Immunofluorescence staining further confirmed Jag1/Notch2 signaling impairment in IEE cells of furcation region in *K14-Cre;Wnt10a*^*fl/fl*^ mice. Conversely, restoring Notch signaling by overexpressing N2ICD partially rescued taurodontism in *K14-Cre;Wnt10a*^*fl/fl*^ mice. These data further confirmed that the Notch signaling acted downstream of *Wnt10a* within IEE cells of HERS during furcation patterning. Former studies indicated that the β-catenin, which functions as the transcription co-activator of Wnt pathway, positively regulated the expression level of Notch2 and Jag1 in epithelial cells.^29,30^ In our study, we validate in dental epithelial cells that *Wnt10a* regulates upstream Notch signaling via active-β-catenin, which provides a novel insight into the Wnt-Notch signaling cascade in tooth root patterning.

Collectively, this study firstly uncovers how HERS cell proper proliferation and oriented division, mediated by Wnt10a-Notch signaling, precisely regulates the patterning of tooth root formation, and highlights the significance of epithelial regulation in root morphogenesis. These findings provide valuable information for bio-root regeneration strategies.

## Materials and methods

### Animal models

Epithelial *Wnt10a* knockout mice, *K14-Cre;Wnt10a*^*fl/fl*^, were generated as previously described.^18^ The mice were housed under specific pathogen-free conditions and used for analysis regardless of sex. Newborn pups were recorded as postnatal stage PN0.5. Genotyping was conducted, on tail samples, using the One Step Mouse Genotyping Kit (Vazyme, Nanjing, China). Primer sequences are listed in Supplementary Table 1. All animal experiments were approved by the Ethics Committee of Peking University Health Science Center (LA2020191).

### Histologic analysis

Mandibles of postnatal mice were dissected on day 0.5, 2.5, 3.5, and 4.5 (PN0.5, PN2.5, PN3.5, and PN4.5), and fixed in 4% paraformaldehyde (PFA) overnight at 4 °C. Subsequently, the samples were decalcified with 10% EDTA for 1 to 4 days, depending on mouse age in days. Decalcified mandibles were dehydrated and embedded in paraffin. The blocks were sectioned (8 μm) using a microtome and mounted on slides (CITOTEST, Nanjing, China) for H&E staining which was performed according to the standard protocol.^41^

### Immunofluorescence assays

Paraffin-embedded sections were prepared as described above (Histologic analysis). The sections were then deparaffinized and rehydrated. Following antigen retrieval (ZSGB-BIO, Beijing, China), sections were blocked for 1 h at room temperature in blocking solution (ZSGB-BIO), and incubated with primary antibodies against E-Cadherin (1:100; ab76319, Abcam, Cambridge, UK), Acetyl-α-Tubulin (1:100; Cell Signaling Technology (CST), Danvers, USA), Cyclin D1 (1:100; CST), Notch2 (1:50; CST), or Jag1 (1:50; CST), overnight at 4 °C. After washing three times with 1x phosphate buffered saline (PSB), sections were then incubated with secondary antibodies (ZSGB-BIO) for 1 h at room temperature. DAPI (ZSGB-BIO) was used to stain cell nucleus. Images were taken (40x), using a confocal microscope (Leica SP8; Wetzlar, Germany).

### EdU proliferation assays

For Keratin-14 (1:100; Abcam) and EdU (5 mg/kg; RiboBio, Guangzhou, China) dual immunofluorescence labeling, PN2.5 and PN3.5 mice (*n* = 5 per stage) were sacrificed 3 h after intraperitoneal EdU injection. Keratin-14 staining was performed as described above (Immunofluorescence assays). After incubated with secondary antibody, the EdU labeling was performed using the using Click-iT Apollo 567 Stain Kit (RiboBio), according to the manufacturer’s instructions and signals were detected using the Leica SP8.

### TUNEL apoptosis analysis

HERS apoptotic cells, in the furcation region, were detected using the TUNEL BrightRed Apoptosis Detection Kit (Vazyme, A113), according to the manufacturer’s instructions.

### Super-resolution microscopy imaging

To further observe the shape and long-axis orientation of the HERS epithelial cells, the mitotic spindle and the precise expression pattern of Notch2, Jag1, and Wnt10a, we photographed the HERS cells at super-resolution using an LSM880 with an Airyscan system (Zeiss, Jena, Germany).

For analysis of cell long axis orientation, we marked and traced the contour of HERS cells to define cell long axis according to the expression pattern of E-Cadherin. Then the angle between the cell long-axis and the horizontal plane was measured for each cell (0° ≤ α ≤ 90°). Radial histograms were generated to demonstrate the central tendency of the cell long-axis direction, using OriginPro (OriginLab Corporation, Northampton, USA). To analyze mitotic spindle orientation, we measured the angle between the long axis of the mitotic spindle and the horizontal plane.

### RNA-Seq analysis and RT-qPCR validation of the dental epithelium

Three pairs of biological replicates (pooling dental epithelium from the five first mandibular molars as one sample) of the RNA samples were collected from PN2.5 *K14-Cre;Wnt10a*^*fl/fl*^ and *Wnt10a*^*fl/fl*^ mice. Samples were sent to Shanghai Majorbio Biopharm Technology Co., Ltd. (Shanghai, China) for mRNA extraction, cDNA library construction, and sequencing with an Illumina Novaseq 6000 platform (San Diego, USA). The raw sequence data was trimmed and quality assessed using SeqPrep (https://github.com/jstjohn/SeqPrep) and Sickle software (https://github.com/najoshi/sickle). Next, the acquired sequences were aligned to the reference genome using HISAT2 (http://ccb.jhu.edu/software/hisat2/index.shtml) software. The fragments per kilobase of exon per million mapped reads (FPKM) method was used to calculate transcript expression levels. RSEM (http://deweylab.biostat.wisc.edu/rsem) was used to quantify gene abundance. Genes differentially expressed between *K14-Cre;Wnt10a*^*fl/fl*^ and *Wnt10a*^*fl/fl*^ samples were detected based on the following criteria: *P*-value < 0.05, and the absolute difference was >20% of *Wnt10a*^*fl/fl*^ group. Heatmap and enrichment analyses of Gene Ontology and KEGG pathways were conducted using the Majorbio Cloud Platform (https://cloud.majorbio.com). The data are available at Gene Expression Omnibus GSE228411. To further confirm Notch signaling gene expression, quantitative RT-PCR was conducted, using the SYBR Green Master Mix (Vazyme) and a 7500 Real-Time PCR detection system (Applied Biosystems, Foster City, CA, USA). β-actin was used as the internal control to normalize expression. Primer sequences are listed in Supplementary Table 2.

### Adenovirus infection and kidney capsule transplantation

A recombinant adenoviral vector (pAdEasy-EF1-MCS-CMV-EGFP), encoding the mouse intracellular domain of Notch2 (N2ICD), was used to overexpress Notch2 and activate Notch signaling. Recombinant adenovirus containing N2ICD (Ad-N2ICD-Over) and an empty adenovirus vector (Ad-Control) were constructed by Shanghai HanBio Technology. To verify the extent of adenovirus infection, the first mandibular molars (*n* = 3) of PN0.5 *Wnt10a*^*fl/fl*^ mice were dissected and infected with Ad-control and Ad-N2ICD-Over. The EGFP signals in the infected molars were detected using an inverted fluorescence microscope. For in vivo rescue experiments, kidney capsule transplantation was performed as previously described.^42^

### Micro-CT analysis

After 21 days of growth in the kidney capsule, the collected mouse kidneys were fixed in 4% PFA overnight at room temperature. Then they were scanned using an Inveon MM system (Siemens, Munich, Germany) to observe the morphology of the root furcation. Images were obtained at 80 kV (voltage), 500 μA (current), 10 μm (pixel size), and 1500 ms (exposure time for each 360 rotational steps). Images were captured using the Inveon MM software.

### Gene silencing and western blot analysis

LS8 cells were seeded in 6-well plates. The next day, after the cells were attached, the corresponding siRNA was transfected using the Ribo FECT^TM^ CP Transfection KIT (RiboBio) according to the instructions. Mouse *Wnt10a* siRNA and negative control siRNA were generated by Tsingke Biotechnology (Beijing, China). The siRNA sequences are upon request.

The cell lysates were harvested and subjected to western blot analysis using primary antibodies including anti-Wnt10a (1:200; Abcam), anti-β-catenin (1:1 000, CST), anti-active-β-catenin (1:1 000, Abcam), anti-N2ICD (1:1 000, CST), anti-Jag1 (1:1 000, CST) or an anti-GAPDH (1:2 000, Abcam). The intensity of each band was visualized using ImageQuant LAS 500 (GE HealthCare) and semi-quantified using ImageJ software (National Institutes of Health). GAPDH was set as normalized control.

### In situ mRNA hybridization of *Wnt10a*

The *Wnt10a* mRNA in situ hybridization was manually carried out by employing PinpoRNA multiplex Fluorescent RNA in-situ hybridization kit (GD Pinpoease Biotech Co.Ltd., Guangzhou, China). Briefly, the sections were deparaffinized and rehydrated, then the endogenous peroxidase was inhibited by Pre-A solution at room temperature. Then we used protease treatment to expose the target RNA molecular, and followed by probe hybridization for 2 h at 40 °C. Signals were amplified sequentially by reaction 1, 2 and 3 as described in manufacturer’s instructions. Finally, a tyramine fluorescent substrate was added and target RNA was labeled by green fluorescent. The signals were detected using an LSM880 with an Airyscan system.

### Statistical analysis

Quantitative results are presented as mean ± SD values. Student’s *t*-test was employed to assess the statistical significance in this article. *P* < 0.05 (*), *P* < 0.01 (**), *P* < 0.001 (***) and *P* < 0.000 1 (****) was considered statistically significant. *P* > 0.05 was considered not significant.

### Supplementary information

SUPPLEMENTAL MATERIAL

## Data Availability

All RNA sequencing data is available via Gene Expression Omnibus, GEO accession GSE228411. Additional data may be made available upon request.
