# Supplementary material for: A Wnt10a-Notch signaling axis controls Hertwig’s epithelial root sheath cell behaviors during root furcation patterning
Source: Int J Oral Sci. 2024 Mar 13;16:25. doi: 10.1038/s41368-024-00288-x (PMC10937922; doi:10.1038/s41368-024-00288-x)
Supplement: Supplementary file 1 — SUPPLEMENTAL MATERIAL [file 41368_2024_288_MOESM1_ESM.docx]

**A Wnt10a-Notch signaling axis controls Hertwig's epithelial root sheath cell behaviors during root furcation patterning**

Kai Sun^1†^, Miao Yu^1†^, Jiayu Wang^1^, Hu Zhao^2^, Haochen Liu^1^, Hailan Feng^1^, Yang Liu^1*^, Dong Han^1*^

^1^Department of Prosthodontics, Peking University School and Hospital of Stomatology & National Center for Stomatology & National Clinical Research Center for Oral Diseases & National Engineering Research Center of Oral Biomaterials and Digital Medical Devices, Beijing, China.

^2^Chinese Institute for Brain Research, Beijing, China.

^*^Corresponding author:

Dong Han: Department of Prosthodontics, Peking University School and Hospital of Stomatology, No.22 Zhongguancun South Avenue, Haidian District, Beijing 100081, PR China. Email: donghan@bjmu.edu.cn.Tel: +86-10-82195393.

Yang Liu: Department of Prosthodontics, Peking University School and Hospital of Stomatology, No.22 Zhongguancun South Avenue, Haidian District, Beijing 100081, PR China. Email: pkussliuyang@bjmu.edu.cn. Tel: +86-10-82195393.

^†^These authors contributed equally to this work

**
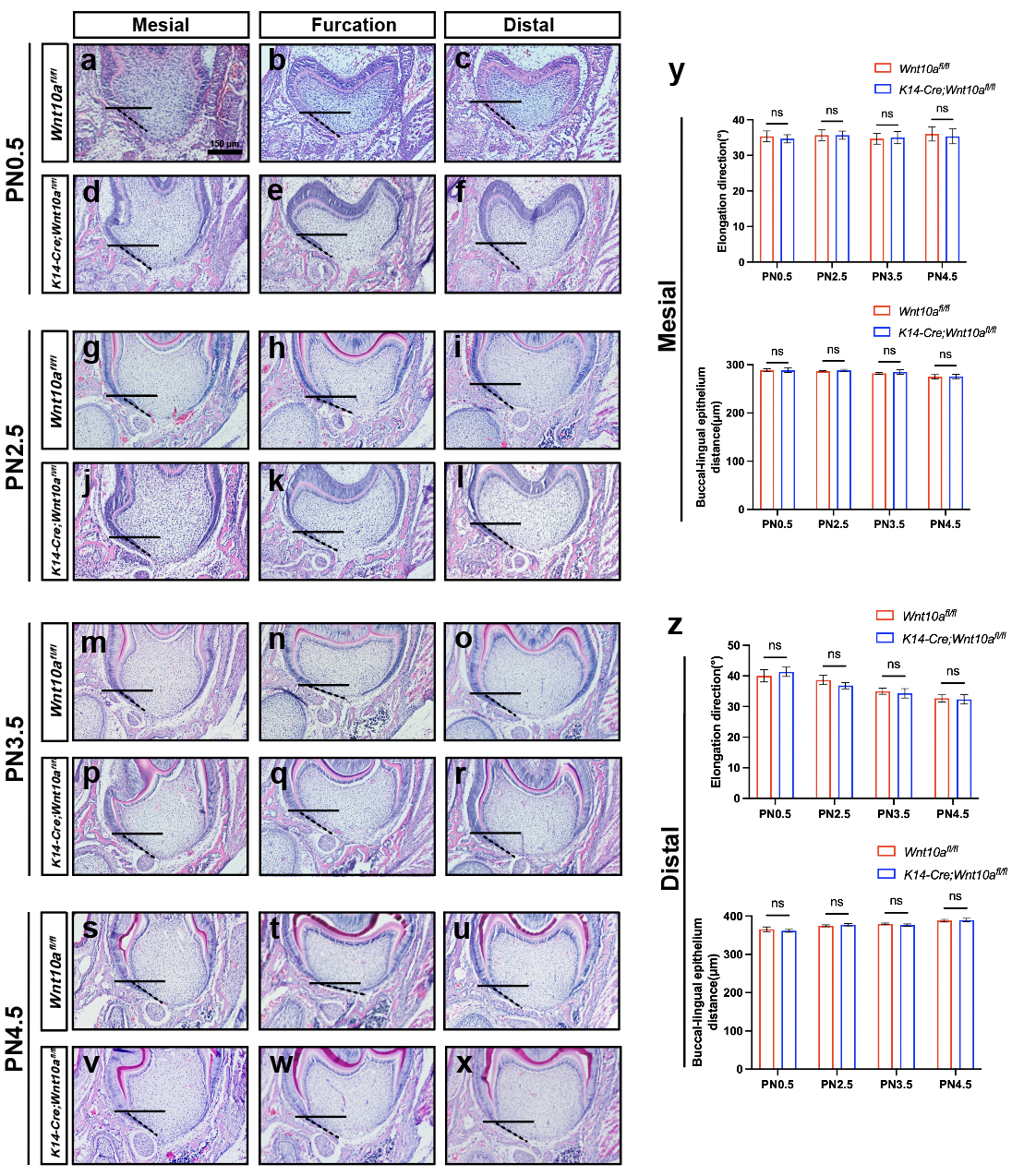
Supplementary Figures and Figure Legends**

**Supplementary Fig. 1** Histology comparison of non-furcation region and furcation region during early-stage postnatal root development of the mandibular first molar. **a-x** Coronal images of H&E-stained mandibular first molars (M1) from *Wnt10a^fl/fl^* and *K14-Cre;Wnt10a^fl/fl^* mice at PN0.5, PN2.5, PN3.5, and PN4.5. Scale bars: 150 μm. **y-z** Bar graphs depicting HERS extension angles **(y)**, and lingual-buccal distance in *Wnt10a^fl/fl^* and *K14-Cre;Wnt10a^fl/fl^* mice **(z)**. n=3 per group. Data are presented as mean ± SD. ns: not significant.

**
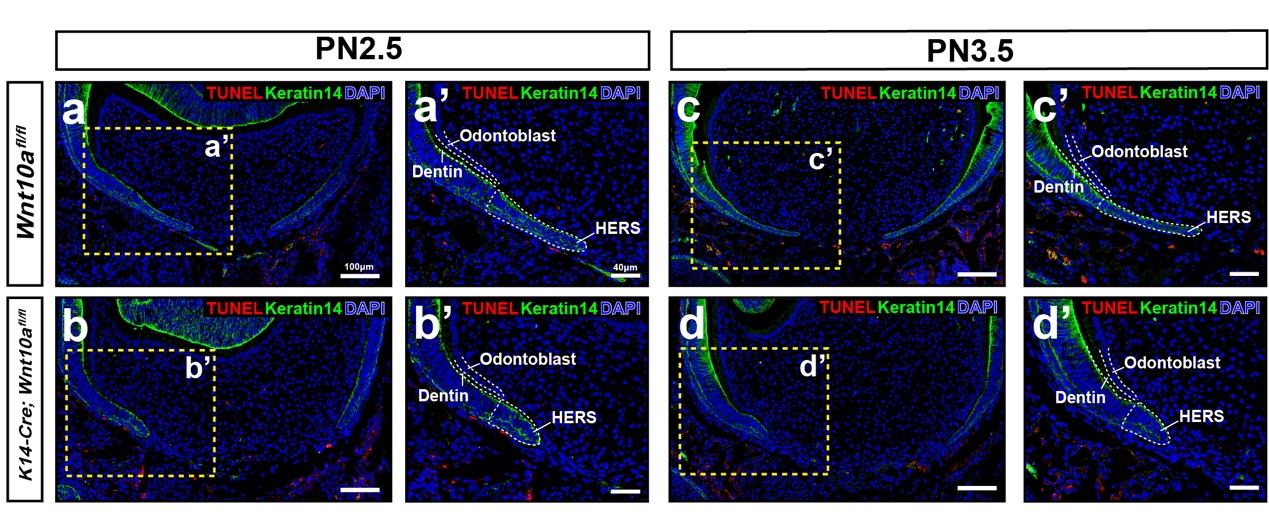
**

**Supplementary Fig. 2** No apoptosis signal was detected in dental epithelial cells of *K14-Cre; Wnt10a^fl/fl^* mice. **a-d** Co-localization of anti-Keratin14 (green), TUNEL (red) and DAPI (blue) shows the apoptosis of dental epithelial cells in the lingual side of the root furcation region of M1 from *Wnt10a^fl/fl^* and *K14-Cre; Wnt10a^fl/fl^* mice at PN2.5 and PN3.5. Scale bars: 100 μm. **a’-d’** Higher magnifications of the yellow dashed line in the images above. White dashed lines outline the HERS cells in the root furcation region for quantification analysis. Scale bars: 40 μm.

**
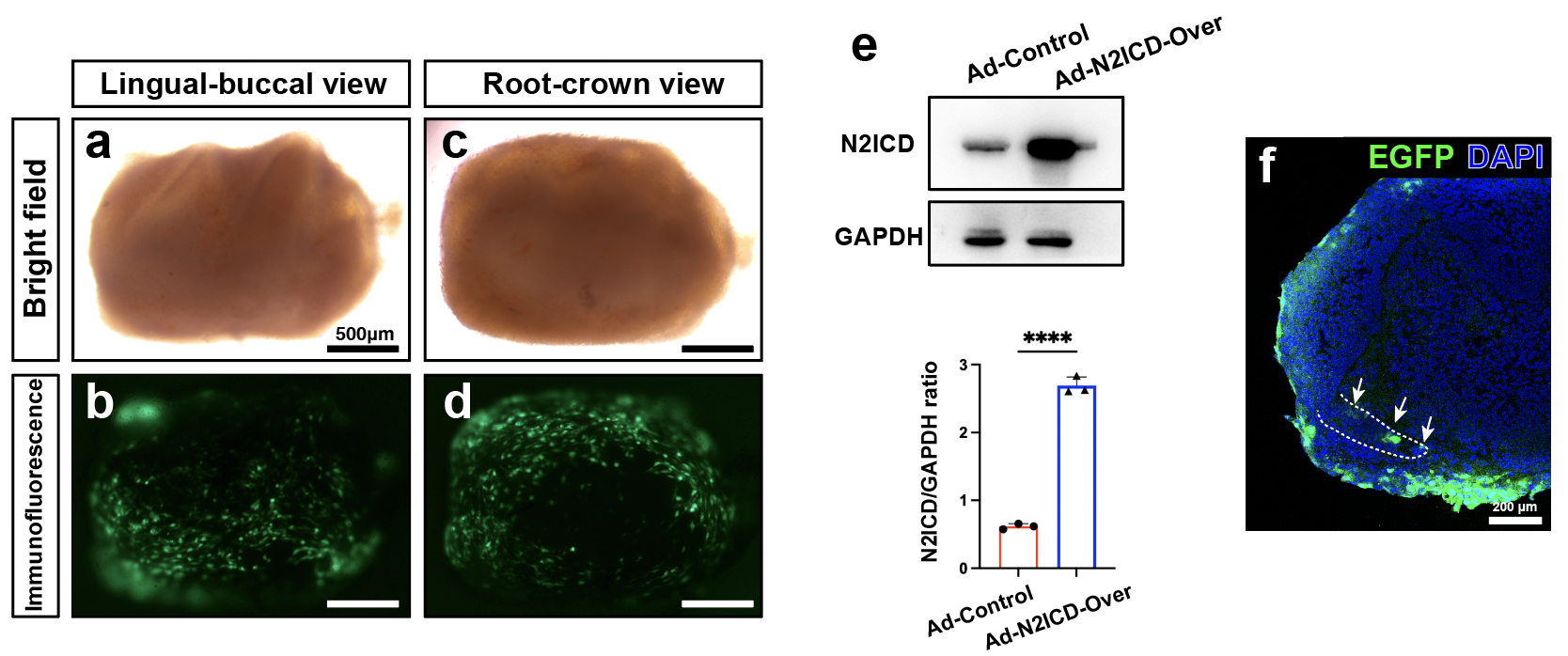
**

**Supplementary Fig. 3** The extent of adenovirus infection and the efficiency of Notch2 overexpression. **a-b** Lingual-buccal (**a**) and root-crown (**b**) views in brightfield of M1 at PN0.5. Scale bars: 500 μm. **c-d** Lingual-buccal (**c**) and root-crown (**d**) views of immunofluorescence image of M1 at PN0.5. Scale bars: 500 μm. **e** Western blot analysis of N2ICD expression level in Ad-Control and Ad-N2ICD-Over M1. Data are presented as mean ± SD. ****P<0.0001. **f** The coronal section of M1 infected by adenovirus. White dashed lines outline HERS cells in the root furcation region. Scale bar: 200 μm.

**
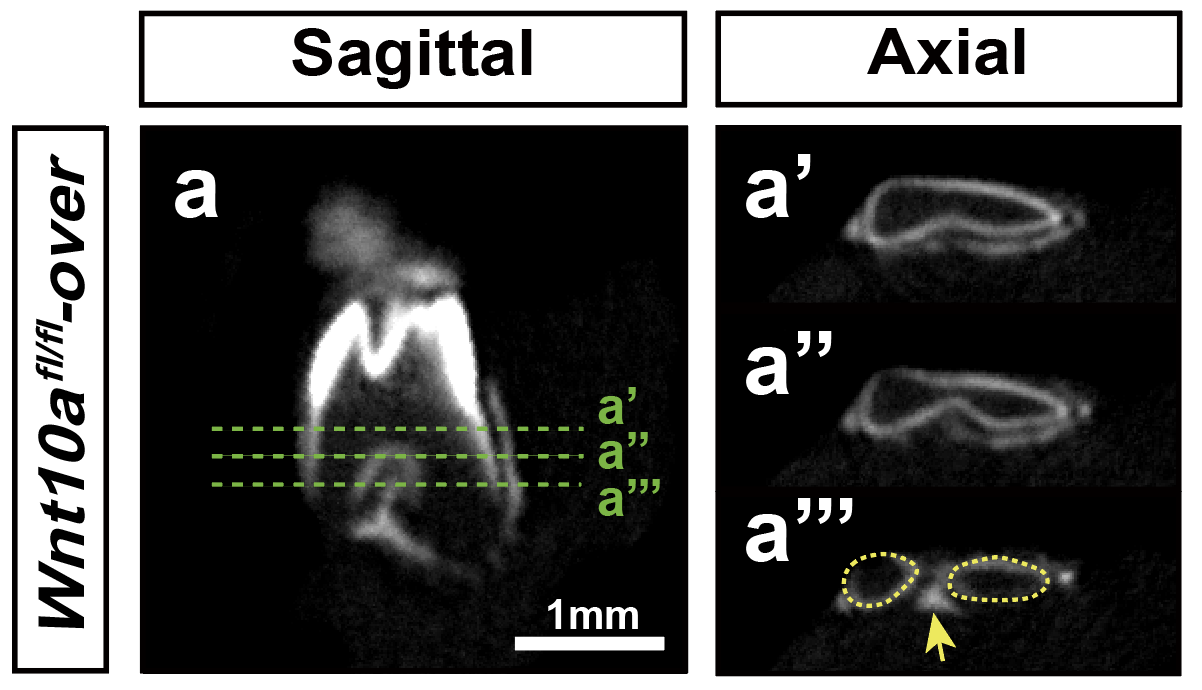
**

**Supplementary Fig. 4** The root furcation of *Wnt10a^fl/fl^* mice shows normal development with overexpression of Notch2. **a** Micro-CT image of *Wnt10a^fl/fl^* molars infected with Ad-N2ICD. **a’-a’’’** Axial views of the upper (**a’**), middle (**a’’)**, and lower (**a’’’**) one-third of the molar roots. Arrows indicate the root furcation region, and yellow dashed lines indicate the contour of each root. Scale bars: 1 mm.

**
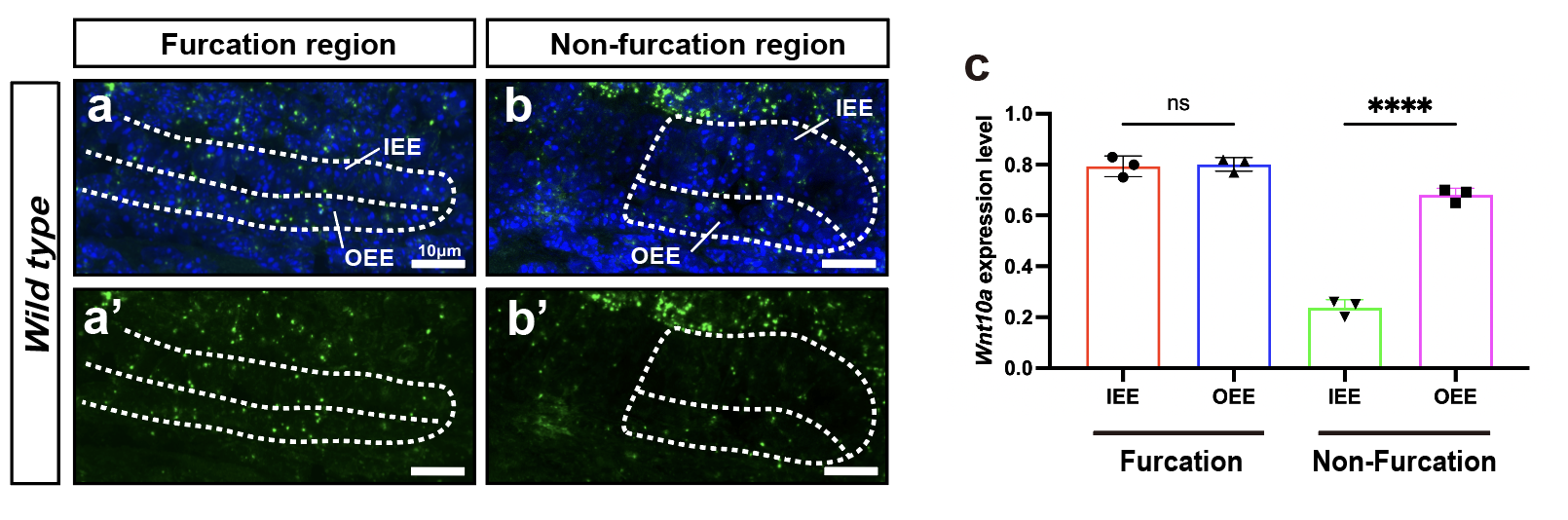
**

**Supplementary Fig. 5** The *Wnt10a* transcript expression pattern in dental epithelium within the furcation region and non-furcation region at PN2.5. **a-b** the mRNA signal of *Wnt10a* (green) and DAPI (blue) shows the *Wnt10a* expression pattern in the lingual HERS of the furcation region and non-furcation region of M1 from *Wnt10a^fl/fl^* mice at PN2.5. white dashed lines outline the IEE and OEE cells, respectively. Scale bars: 10 μm. **c** Expression level of *Wnt10a* in IEE or OEE cells. Data are presented as mean ± SD. ****P<0.0001.

**
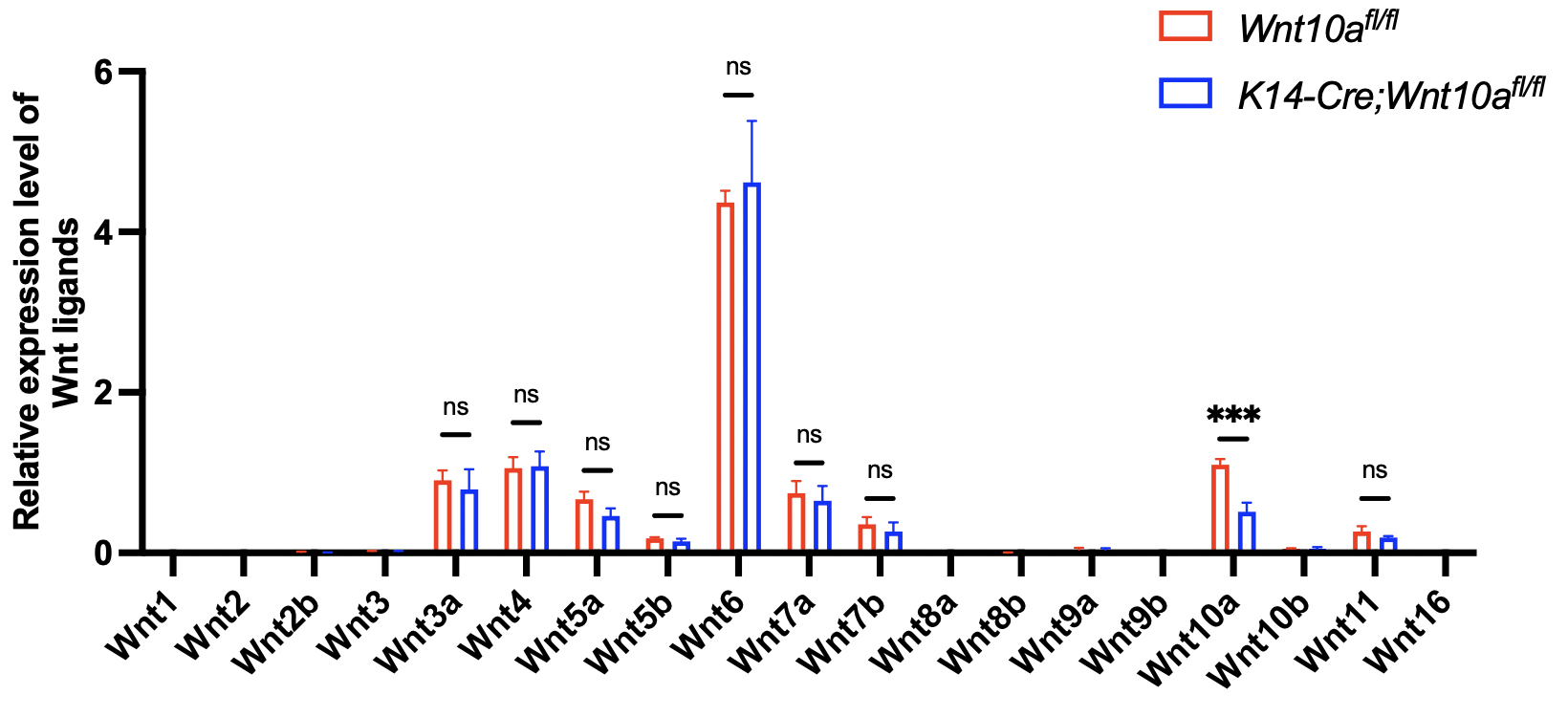
**

**Supplementary Fig. 6** The expression level of Wnt signaling ligands in the M1 dental epithelium of *Wnt10a^fl/fl^* and *K14-Cre;Wnt10a^fl/fl^* mice at PN2.5. *Wnt4* expression in *Wnt10a^fl/fl^* samples was set to 1. n=3 per group.

**Supplementary Tables**

**Supplementary Table 1. Primers for genotyping**

| **Name** | **Primer sequence** |
| --- | --- |
| *Wnt10a* pimer 1 | CGACACGTATAACTTCGTAT |
| *Wnt10a* pimer 2 | TCTCTCTGACCCTAAGGAGC |
| *Wnt10a* pimer 3 | TCACTGTCACTCGAGGCAA |
| *K14-Cre* primer 1 | GCCTGCATTACCGGTCGATGC |
| *K14-Cre* primer 2 | CAGGGTGTTATAAGCAATCCC |

**Supplementary Table 2. Primers for qPCR**

| **Gene** | **Forward** | **Reverse** |
| --- | --- | --- |
| *Notch1* | CCCTTGCTCTGCCTAACGC | GGAGTCCTGGCATCGTTGG |
| *Notch2* | GACTGCCAATACTCCACCTCT | CCATTTTCGCAGGGATGAGAT |
| *Notch3* | AGTGCCGATCTGGTACAACTT | CACTACGGGGTTCTCACACA |
| *Notch4* | CTCTTGCCACTCAATTTCCCT | TTGCAGAGTTGGGTATCCCTG |
| *Jagged1* | CCTCGGGTCAGTTTGAGCTG | CCTTGAGGCACACTTTGAAGTA |
| *Jagged2* | CAATGACACCACTCCAGATGAG | GGCCAAAGAAGTCGTTGCG |
| *Dll1* | CAGGACCTTCTTTCGCGTATG | AAGGGGAATCGGATGGGGTT |
| *Dll3* | CTGGTGTCTTCGAGCTACAAAT | TGCTCCGTATAGACCGGGAC |
| *Dll4* | TTCCAGGCAACCTTCTCCGA | ACTGCCGCTATTCTTGTCCC |
